# Exploring metabolite-mediated links between lipidome and deep vein thrombosis: Insights from Mendelian randomization analysis

**DOI:** 10.1097/MD.0000000000041783

**Published:** 2025-03-07

**Authors:** Zhenyu Liu, Hang Ma, Lin Zhang, Xiaocheng Xu, Shuai Su, Xiangbiao He

**Affiliations:** a Department of Urology, The First Affiliated Hospital of Chongqing Medical University, Chongqing, China; b Department of Rheumatology, The First Affiliated Hospital of Zhengzhou University, Zhengzhou, Henan Province, China.

**Keywords:** deep vein thrombosis, lipidome, mediator, Mendelian randomization, metabolites

## Abstract

The aim was to investigate the causal relationship between the lipidome and deep vein thrombosis (DVT) while identifying and quantifying the role of metabolites as potential mediators. Two-sample Mendelian randomization (MR) analysis of lipid species (n = 7174) and DVT (6767 cases and 330,392 controls) was performed using pooled data from genome-wide association studies. In addition, we quantified the proportion of metabolite-mediated lipidomic effects on DVT using 2-step MR. Phosphatidylcholine (18:0_18:2) levels mined from 179 lipids using MR analysis reduced the risk of DVT (odds ratio [OR]: 0.997; 95% CI: 0.996–0.999; *P* = 4.25 × 10^−4^; false discovery rate [FDR] = 0.013). Octadecadienedioate (C18:2-DC) levels increased with the increasing phosphatidylcholine (18:0_18:2) levels (OR: 1.087, 95% confidence interval [CI]: [1.024, 1.154], *P* = .006). Octadecadienedioate (C18:2-DC) levels mined from 1400 metabolites using MR analysis reduced the DVT risk (OR: 0.997; 95% CI: [0.996, 0.999], *P* = 6.11 × 10^−6^; FDR = 8.55 × 10^−3^). The proportion of the predicted genes for phosphatidylcholine (18:0_18:2) levels mediated by octadecadienedioate (C18:2-DC) levels was 7.8%. This study identified octadecadienedioate (C18:2-DC) levels as a potential mediator of the causal relationship between phosphatidylcholine (18:0_18:2) levels and DVT, which provides direction for the future investigation of DVT; however, further research on other potential mediators is still needed.

## 1. Introduction

Deep vein thrombosis (DVT) is classified as venous thromboembolism (VTE) and is a relatively common chronic disease.^[1]^ In addition to well-known blood flow disorders, hypercoagulability, and vessel wall changes, inflammation also plays an important role in the development of DVT.^[2,3]^ Several clinical conditions associated with inflammation, such as systemic infections, sepsis, cancer, trauma, and surgery, are associated with an increased risk of DVT.^[4]^ Oral anticoagulants are the first-line treatment for almost all cases of venous thrombosis.^[5]^ Additionally, a meta-analysis has shown that statins are protective against DVT, and rosuvastatin significantly reduces the risk of VTE compared to other statins.^[6]^ Another study showed that lipoprotein(a) is a risk factor for venous thrombosis and is significantly associated with an increased risk of VTE.^[7]^ Jiang et al^[8]^ showed that altered levels of C5 carnitine and diacylglycerol are significantly associated with VTE. Another animal study demonstrated that adenosine, adenine, citric acid, succinic acid, and fumarate levels were reduced to varying degrees in the serum of DVT mice, whereas L-carnitine levels were higher. Several metabolites, including acetylcarnitine, adenosine, and ceramide, are present in the vein walls of mice.^[9]^ Obi et al^[10]^ showed that glutamine, proline, and phenylalanine levels increased in aged mice with venous thrombosis. Fraser et al^[11]^ identified 21 plasma metabolites using plasma metabolomic analysis, which contained 12 lipids that could be used as biological markers of stable VTE. The results of these studies indicate a seemingly inextricable link between lipids, metabolites, and venous thrombosis.

According to estimates, lipids are characterized by structural diversity and are present in significant quantities in a wide range of species.^[12]^ An increasing number of patients with metabolic and cardiovascular diseases require more detailed lipid analysis for the diagnosis and monitoring of drug efficacy.^[13]^ The 179 lipids included in this study were obtained from a recent study by Ottensmann et al,^[14]^ who used univariate and multivariate genome-wide analyses to reveal genetic links between disease and lipids. Metabolites are the products of metabolic reactions and are influenced by a variety of factors, including genetics, diet, gut microbes, and disease.^[15,16]^ The 1400 metabolites included in this study were obtained from Chen et al.^[17]^ Through a series of large-scale genome-wide association studies (GWAS), they inferred causal relationships between metabolite levels and multiple traits or diseases. Based on existing studies, this study explored the possible causal relationship between lipidome, metabolites, and DVT by means of Mendelian randomization (MR) analysis and identified potential metabolites that may be used as early diagnostic and therapeutic targets.

## 2. Materials and methods

### 2.1. Study design

This study explored the reciprocal causal relationship between lipid species and DVT using a 2-sample bidirectional MR. Here are 3 key assumptions that must be consistently followed when conducting MR analyses: Relevance assumption: instrumental variables (IVs) and exposures are highly correlated with each other. Independence assumption: IVs are not associated with confounders. Exclusion restriction assumption: IVs affect outcome only through exposure and are not directly related to outcome. All data used in this study are publicly available and have been approved by the institutional review boards of the respective studies. All the generated results are presented in the article and supplementary documents.

### 2.2. Data sources

The GWAS participants were of European origin. We obtained GWAS data for lipid species from a recent study by Ottensmann et al,^[14]^ which included 771 Finnish individuals; the data are also available from http://ftp.ebi.ac.uk/pub/databases/gwas/summary_statistics/GCST90277001-GCST90278000/. DVT data were obtained from the UK Biobank, including 6767 cases and 330,392 controls, and are available from https://gwas.mrcieu.ac.uk/. The study by Chen et al^[17]^ obtained genetic associations of plasma metabolites, for which data for European populations are available from http://ftp.ebi.ac.uk/pub/databases/gwas/summary_statistics/GCST90199001-GCST90200000/.

### 2.3. Selection of instrumental variables (IVs) and data harmonization

First, we selected single nucleotide polymorphisms (SNPs) as candidate IVs based on the criterion of *P* < 1 × 10^−5^ in a 10,000 kb window. Meanwhile, independent SNPs were required to exhibit low association with other SNPs in the region (*r*^2^ < 0.001). Second, the effective SNPs were extracted from the outcome GWAS dataset using a filtering criterion of minor allele frequency (MAF) > 0.01. Next, valid IVs were obtained after harmonizing the exposure and outcome effects and removing SNPs with an *F* statistic < 10 or a failure to harmonize. To calculate the *F* value, we used the latest and most accurate methodology: *F* = *R*^2^(N − *K* − 1)/*K*(1 − *R*^2^), where *R*^2^ is the cumulative variance of the exposure, *K* is the total number of IVs, and N is the total number of samples. It is generally accepted that an *F* statistic > 10 is sufficient to prevent weak instrumental bias.^[18,19]^ In addition, we used PhenoScanner (http://www.phenoscanner.medschl.cam.ac.uk/) to search for relationships between IVs and phenotypes, and then removed IVs associated with confounders. Statistical methods for controlling the false discovery rate (FDR) have become popular and powerful tools for controlling the error rate. We used the Benjamini–Hochberg method to adjust *P* values for the analysis of the Mendelian randomization with multiple exposures^[20,21]^

### 2.4. Statistical analysis

All statistical analyses for this study were performed in R v4.2.3 using the “TwoSampleMR,” “VariantAnnotation,” “gwasglue,” “ieugwasr,” “grid,” “readr,” “forestploter,” and “p.value” R packages. Two-sample Mendelian randomization was performed using the “TwoSampleMR” package.

### 2.5. Primary analysis and mediation analysis

**Figure 1.**
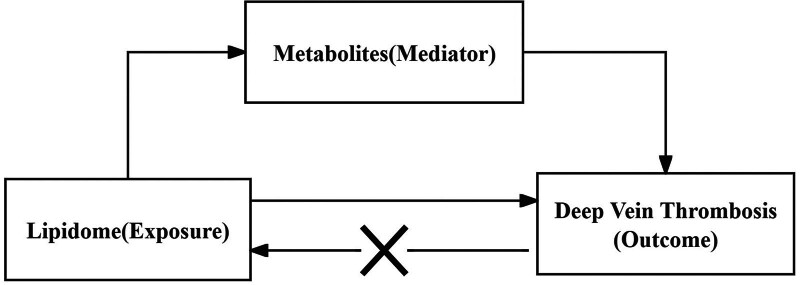
The basic principles of mediated Mendelian analysis.

**Figure 2.**
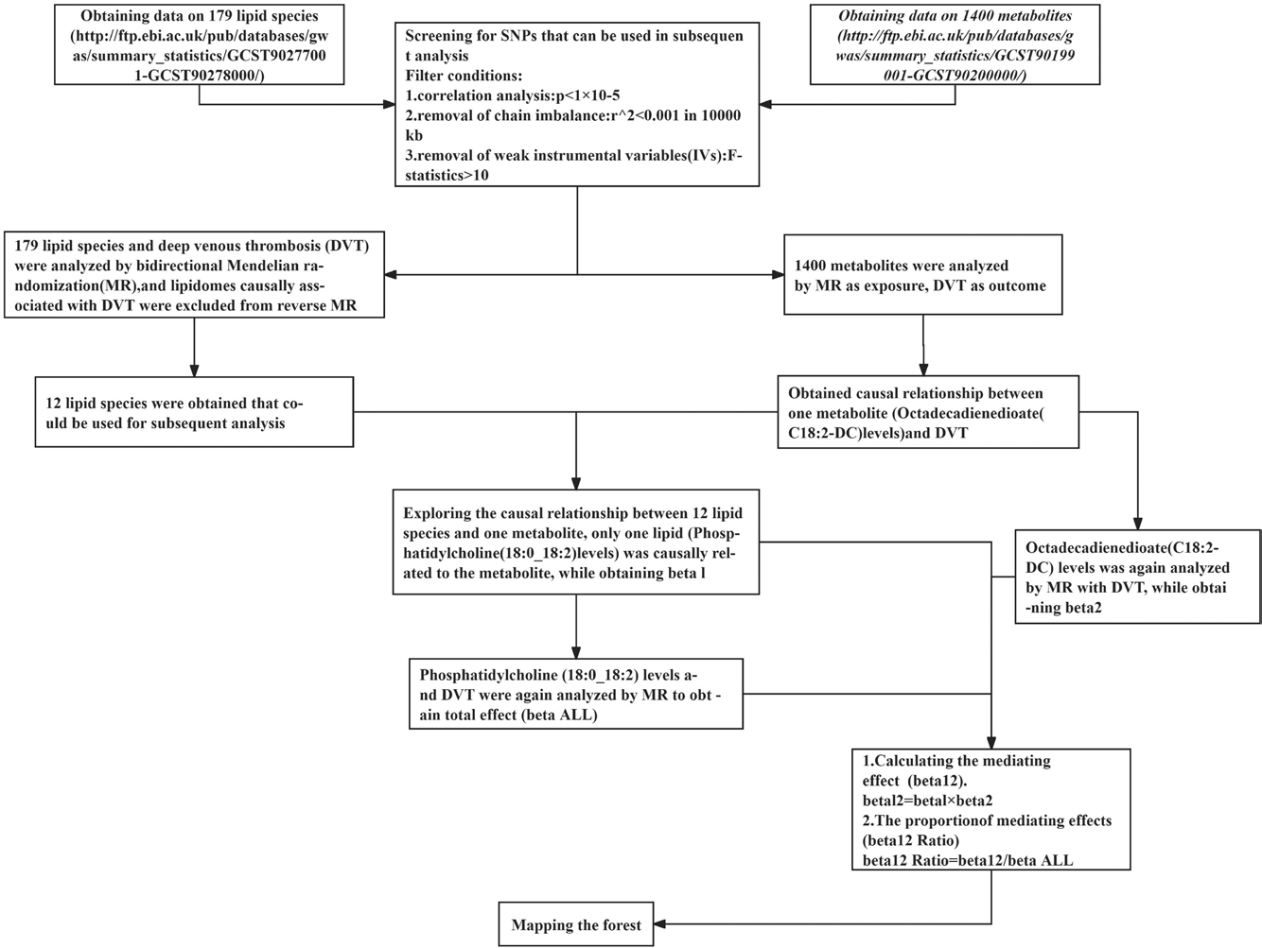
The flowchart of this study. DVT = deep vein thrombosis, MR = Mendelian randomization.

Figure 1 shows the basic principles of the mediated Mendelian randomization analysis. Figure 2 presents a flowchart of this study. Initially, we explored the causal relationship between 179 lipid species and DVT (Supplementary file 1, Supplemental Digital Content, http://links.lww.com/MD/O474). MR assessment of effective IVs was performed using inverse variance weighting (IVW), weighted mode, simple mode, weighted median, and MR-Egger regression. We evaluated heterogeneity using Cochran’s *Q* test and funnel plots, conducted pleiotropy analysis using the MR-Egger intercept, and visualized the MR results. After filtering out the disease-related lipidome based on the FDR of the IVW method, the *P* value of pleiotropy, and the odds ratio (OR) direction of the 5 methods (Supplementary file 2, Supplemental Digital Content, http://links.lww.com/MD/O474), we performed reverse MR. We used the disease-related lipidome as the outcome and DVT as the exposure to eliminate the lipids causally related to DVT, and the remaining lipids were used for subsequent analyses (Supplementary file 3, Supplemental Digital Content, http://links.lww.com/MD/O474). We then explored the causal relationship between 1400 metabolites and DVT (Supplementary file 4, Supplemental Digital Content, http://links.lww.com/MD/O474) and screened for metabolites associated with the disease (Supplementary file 5, Supplemental Digital Content, http://links.lww.com/MD/O474).

We further explored whether metabolites mediate the causal pathway from the lipidome to DVT using 2-step MR. The beta value for the lipidome on metabolite causality was defined as beta 1. The beta value for the causality of the metabolites in DVT was defined as beta 2. The beta value of the lipidome in DVT was defined as beta ALL. The mediating effect was equal to beta 1 × beta 2, and the direct effect was equal to the total effect minus the mediating effect^[22]^ The proportion of the mediating effect was equal to the mediating effect/total effect^[23]^

### 2.6. Ethical statement

Publicly available databases were utilized for this study. Each individual study in GWAS was approved by the appropriate institutional review board, and participants or their authorized representatives provided informed consent.

## 3. Results

### 3.1. Association of lipidome with metabolites

**Figure 3.**
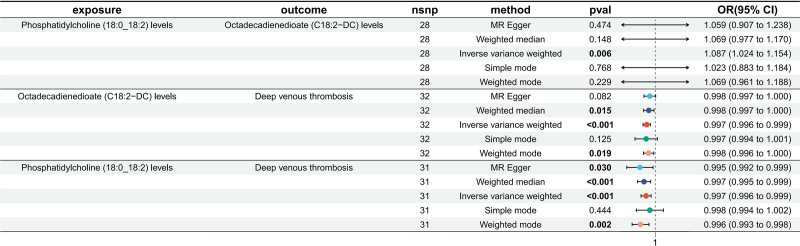
Forest plot to visualize the causal effect of octadecadienedioate (C18:2-DC) levels with Phosphatidylcholine (18:0_18:2) levels and DVT. DVT = deep vein thrombosis, MR = Mendelian randomization.

First, we filtered out 15 lipid species causally associated with DVT (Supplementary file 2, Supplemental Digital Content, http://links.lww.com/MD/O474). Following reverse MR analysis, we excluded 3 of these lipid species, yielding a final set of 12 lipid species for subsequent analysis (Supplementary file 3, Supplemental Digital Content, http://links.lww.com/MD/O474). Only 1 of the 12 lipid species (phosphatidylcholine (18:0_18:2) levels) had a causal relationship with the metabolite (octadecadienedioate (C18:2-DC) levels). A total of 28 SNPs were included for MR analysis as follows: IVW OR: 1.087, 95% CI: [1.024, 1.154], *P* = .006 (Fig. 3, Supplementary file 6, Supplemental Digital Content, http://links.lww.com/MD/O474). Except for IVW, the other 4 methods did not show statistical significance; however, their OR values aligned in the same direction, with neither heterogeneity nor multiplicity showing statistical significance (Supplementary file 6, Supplemental Digital Content, http://links.lww.com/MD/O474), suggesting a causal relationship between phosphatidylcholine (18:0_18:2) levels and octadecadienedioate (C18:2-DC) levels. Supplementary file 7, Supplemental Digital Content, http://links.lww.com/MD/O476 shows the scatter plot, forest plot, funnel plot, and leave-one-out analysis of the MR analysis to demonstrate its stability.

### 3.2. Association of metabolites with DVT

Among the 1400 metabolites, we screened for a causal relationship between octadecadienedioate (C18:2-DC) levels and DVT (Supplementary file 5, Supplemental Digital Content, http://links.lww.com/MD/O474). A total of 32 SNPs were included in the study, and the IVW method was the main analysis method. The OR of IVW was 0.997 (95% CI: [0.996, 0.999], *P* = 6.11 × 10^−6^, FDR = 8.55 × 10^−3^; Fig. 3, Supplementary file 8, Supplemental Digital Content, http://links.lww.com/MD/O475). The directions of the ORs for all 5 methods were consistent, and the FDR for IVW was <0.05, while neither heterogeneity nor pleiotropy showed statistically significant results (Supplementary file 8, Supplemental Digital Content, http://links.lww.com/MD/O475). The visualization of the MR analysis for the relationship between octadecadienedioate (C18:2-DC) levels and DVT is shown in Supplementary file 9, Supplemental Digital Content, http://links.lww.com/MD/O476. The results demonstrate the robustness of our findings, suggesting that octadecadienedioate (C18:2-DC) levels may serve as a protective factor against DVT.

### 3.3. Association of lipidome with DVT

We identified 15 lipid species that were causally associated with DVT. After eliminating 3 lipid species exhibiting reverse causality with DVT, only phosphatidylcholine (18:0_18:2) levels were causally related to octadecadienedioate (C18:2-DC) levels. Therefore, we performed an MR analysis of phosphatidylcholine (18:0_18:2) levels using DVT to determine the total effect. The results revealed that phosphatidylcholine (18:0_18:2) levels were a protective factor against DVT, and the OR of the IVW method was 0.997 (95% CI: 0.996–0.999, *P* = 4.25 × 10^−4^, FDR = 0.013). Among the remaining 4 methods, only the simple mode failed to show statistical significance (Fig. 3, Supplementary file 10, Supplemental Digital Content, http://links.lww.com/MD/O475). Additionally, neither the heterogeneity nor the pleiotropy results were statistically significant (Supplementary file 10, Supplemental Digital Content, http://links.lww.com/MD/O475). The scatter plot, forest plot, funnel plot, and leave-one-out analysis all exhibited consistent results (Supplementary file 11, Supplemental Digital Content, http://links.lww.com/MD/O476).

### 3.4. Proportion of the association between lipidome and DVT mediated by metabolites

**Figure 4.**
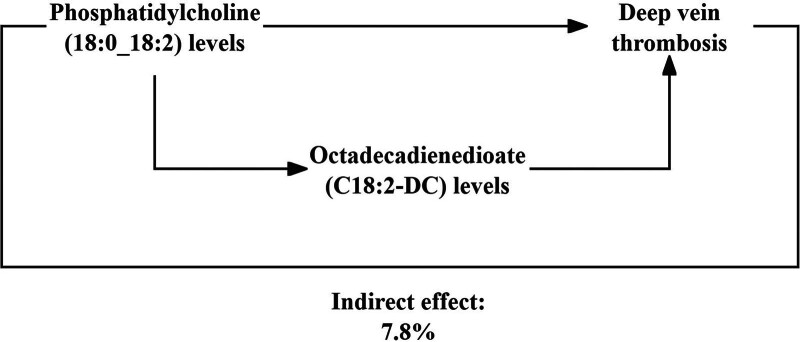
Schematic diagram of octadecadienedioate (C18:2-DC) levels mediation effect.

We analyzed the octadecadienedioate (C18:2-DC) levels as a mediator of the pathway from phosphatidylcholine (18:0_18:2) levels to DVT. We found that phosphatidylcholine (18:0_18:2) levels were associated with increased octadecadienedioate (C18:2-DC) levels, which, in turn, were associated with a decreased risk of DVT. As shown in Figure 4, our study demonstrated that octadecadienedioate (C18:2-DC) levels accounted for 7.8% of the reduction in DVT risk associated with phosphatidylcholine (18:0_18:2) levels.

## 4. Discussion

Thrombosis is a disease that jeopardizes the life and health of human beings and imposes a heavy burden on the world’s healthcare systems. It primarily develops from a range of traumatic and non-traumatic injuries or other diseases, leading to intravascular damage and subsequent hemostatic responses.^[24]^ Thrombosis can occur in the arteries and veins at all levels of the vascular system.^[25]^ VTE, which can be further categorized into DVT and pulmonary embolism, is a common, chronic condition, the causes of which include major surgeries and cancer, but most cases are unprovoked.^[1]^ A relationship between lipids and venous thrombosis has been demonstrated.^[26–28]^ However, most of these studies were on classical lipids (low-density lipoproteins, high-density lipoproteins, triglycerides) and venous thrombosis; they were also observational, and the results may have been affected by confounding factors. Although 2 recent MR studies demonstrated no significant genetic association between classical lipids and venous thrombosis, they were limited to classical lipids and did not delve deeply into the underlying mechanisms that may exist between lipids and venous thrombosis.^[29,30]^ Our study aimed to determine the causal relationship between 179 lipid species and DVT. Using the existing GWAS database, we applied MR analysis to study the relationship between lipids and DVT and to determine whether the relationship was mediated through metabolites. Our findings identified a potential association between phosphatidylcholine (18:0_18:2) levels and a reduced risk of DVT, of which 7.8% was mediated by octadecadienedioate (C18:2-DC) levels.

To date, we are the first to investigate the causal relationship between metabolite-mediated lipidome and DVT using mediated MR analysis, using octadecadienedioate (C18:2-DC) levels as a mediator. A previous animal study showed that phosphatidylcholine levels were reduced by an average of 1.8-fold in DVT mice.^[9]^ Another recent study showed a significant decrease in the concentration of phosphatidylcholines in a DVT rat model, with phosphatidylcholine (20:6_20:2) being the most significant.^[31]^

Octadecadienedioate is a dicarboxylic acid dianion obtained by the deprotonation of 2 carboxyl groups of octadecanedioic acid. There is a lack of evidence of a correlation between octadecadienedioate (C18:2-DC) levels and DVT; however, some studies have directly or indirectly demonstrated a relationship between linoleic acid (LA) and thrombosis. Chen et al^[32]^ found that coupling LA to low-molecular weight heparin through ester bonding enhanced the therapeutic efficacy over low-molecular weight heparin alone in eliminating thrombosis and subsequent DVT development in pregnant rats. Tao et al^[33]^ demonstrated that LA has antiplatelet and antithrombotic effects both in vivo and in vitro. Another MR study demonstrated that higher LA levels were associated with a decreased risk of VTE, especially DVT.^[34]^

Hydroxyoctadecadienoic acid is produced from LA via chemical reactions.^[35]^ High-density lipoproteins enriched in phosphatidylcholine species containing Hydroxyoctadecadienoic acid have been shown to inhibit platelet aggregation.^[36]^ Phosphatidylcholine and LA also exert anti-inflammatory effects.^[37,38]^ Although inflammation plays an important role in the development of DVT, it is reasonable to suspect that octadecadienedioate (C18:2-DC) levels act as a mediator of the causal relationship between phosphatidylcholine (18:0_18:2) levels and DVT. Based on the current study, clinically, we can consider monitoring phosphatidylcholine (18:0_18:2) levels and/or octadecadienedioate (C18:2-DC) in patients at high risk of DVT to detect DVT as early as possible, to prevent further injuries such as pulmonary embolism. However, there are many kinds of lipids and metabolites, and more studies should be completed as soon as possible to improve the efficacy of predicting DVT.

It is important to note that this study had several limitations. First, we only included a study population from Europe, reducing the generalizability of this study. Second, we used a loose threshold, and although we later used the FDR for *P* value correction, there was still a risk of false positives. Third, we used summary-level statistics rather than individual-level data. As a result, we were unable to further explore the causal relationships between subgroups, such as females and males. Fourth, our results indicate that octadecadienedioate (C18:2-DC) levels mediated a relatively low genetic prediction rate of 7.8%. Therefore, further studies are required to quantify the effects of other mediators. Fifth, the variety of lipids in our selected dataset remained limited compared to those previously identified.

## 5. Conclusion

In conclusion, this study demonstrated a causal relationship between phosphatidylcholine (18:0_18:2) levels and DVT and identified octadecadienedioate (C18:2-DC) levels as a potential mediator of their relationship. Our findings provide a promising direction for researchers to further explore the mechanism of DVT. However, due to the wide variety of lipids and serum metabolites, most of the effects of phosphatidylcholine (18:0_18:2) levels on DVT are still unclear, and more studies on other potential mediators are needed.

## Acknowledgments

We acknowledge the participants and investigators of the FinnGen study.

## Author contributions

**Conceptualization:** Zhenyu Liu, Hang Ma, Lin Zhang, Xiaocheng Xu, Shuai Su, Xiangbiao He.

**Data curation:** Zhenyu Liu, Hang Ma, Lin Zhang, Xiaocheng Xu, Xiangbiao He.

**Formal analysis:** Zhenyu Liu, Hang Ma, Lin Zhang, Shuai Su, Xiangbiao He.

**Funding acquisition:** Shuai Su.

**Investigation:** Zhenyu Liu, Hang Ma, Xiangbiao He.

**Methodology:** Zhenyu Liu, Hang Ma, Shuai Su, Xiangbiao He.

**Project administration:** Zhenyu Liu, Hang Ma, Shuai Su, Xiangbiao He.

**Resources:** Zhenyu Liu, Hang Ma, Shuai Su, Xiangbiao He.

**Software:** Zhenyu Liu, Hang Ma, Lin Zhang, Xiaocheng Xu, Shuai Su, Xiangbiao He.

**Supervision:** Zhenyu Liu, Hang Ma, Lin Zhang, Xiaocheng Xu, Shuai Su, Xiangbiao He.

**Validation:** Zhenyu Liu, Hang Ma, Lin Zhang, Xiaocheng Xu, Shuai Su, Xiangbiao He.

**Visualization:** Zhenyu Liu, Hang Ma, Lin Zhang, Shuai Su, Xiangbiao He.

**Writing – original draft:** Zhenyu Liu, Hang Ma, Lin Zhang, Xiaocheng Xu.

**Writing – review & editing:** Zhenyu Liu, Hang Ma, Shuai Su, Xiangbiao He.

## Supplementary Material
