# Supplementary material for: Exploring metabolite-mediated links between lipidome and deep vein thrombosis: Insights from Mendelian randomization analysis
Source: Medicine (Baltimore). 2025 Mar 7;104(10):e41783. doi: 10.1097/MD.0000000000041783 (PMC11902998; doi:10.1097/MD.0000000000041783)
Supplement: Supplementary file 2 [file medi-104-e41783-s002.pdf]

Supplementary file 7: Visualization of MR result for Phosphatidylcholine (18:0_18:2) levels on Octadecadienedioate (C18:2-DC) levels.

Visualization of the MR Results (*Phosphatidylcholine (18:0_18:2) levels on Octadecadienedioate (C18:2-DC) levels*)

Scatter plot

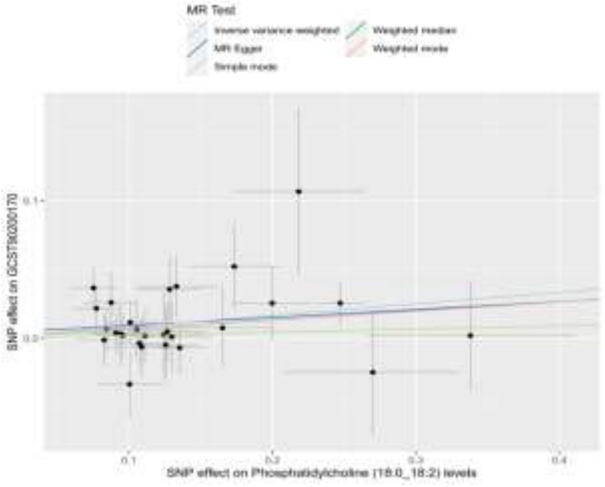

Funnel plot

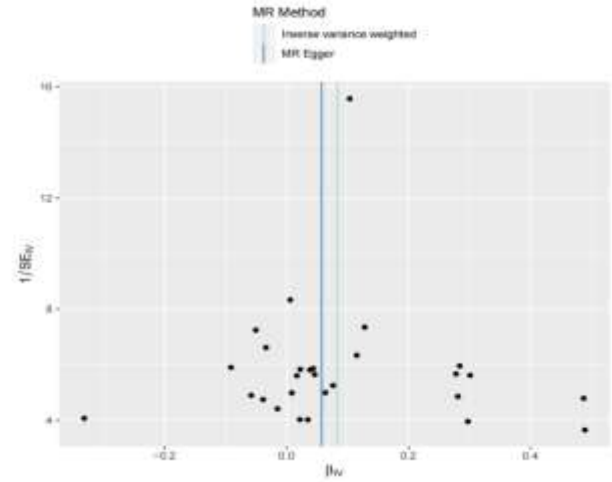

Supplementary file 9: Visualization of MR result for Octadecadienedioate (C18:2-DC) levels on Deep venous thrombosis (DVT).

Leave-one-out analysis

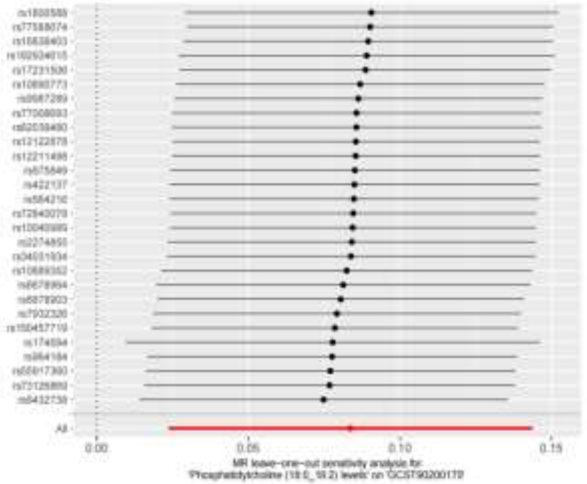

Forest plot

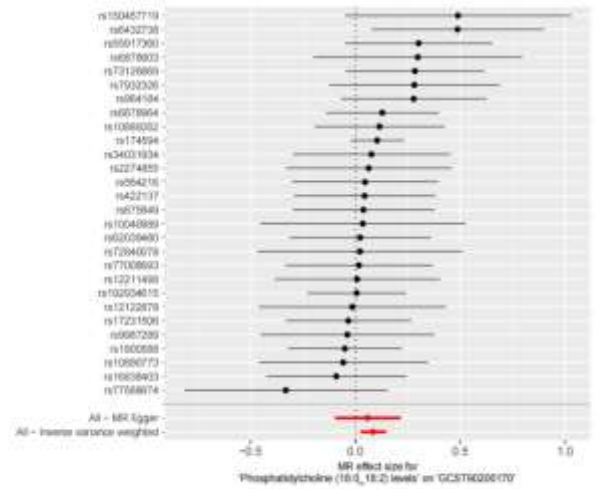

Visualization of the MR Results (*Octadecadienedioate (C18:2-DC) levels on DVT*)

Scatter plot

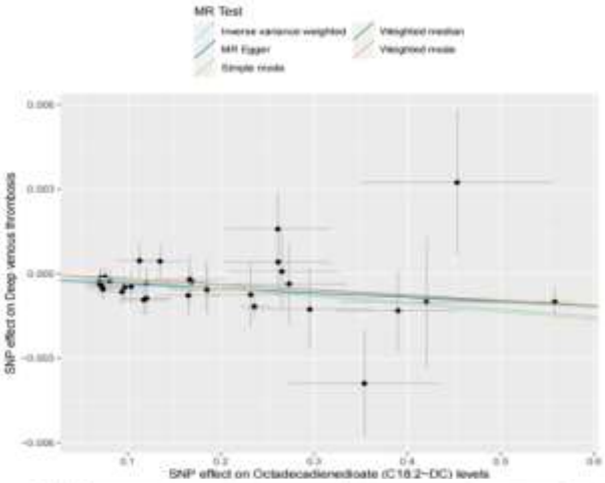

Funnel plot

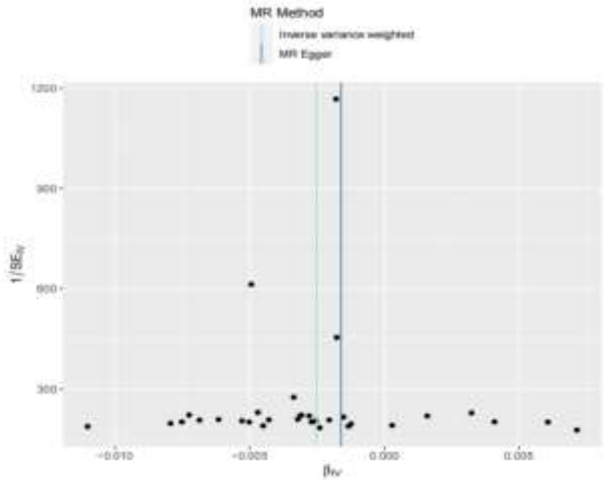

Forest plot

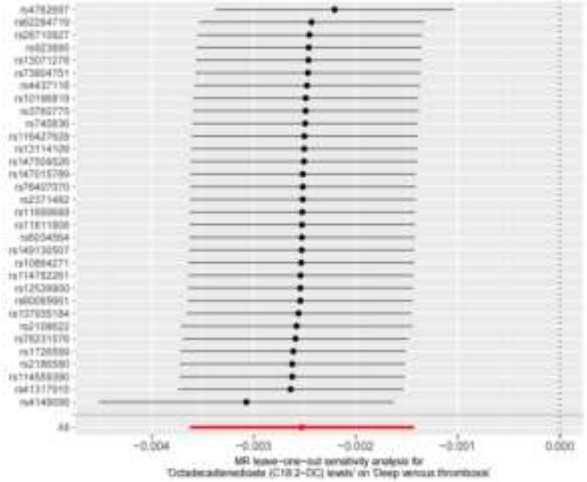

Leave-one-out analysis

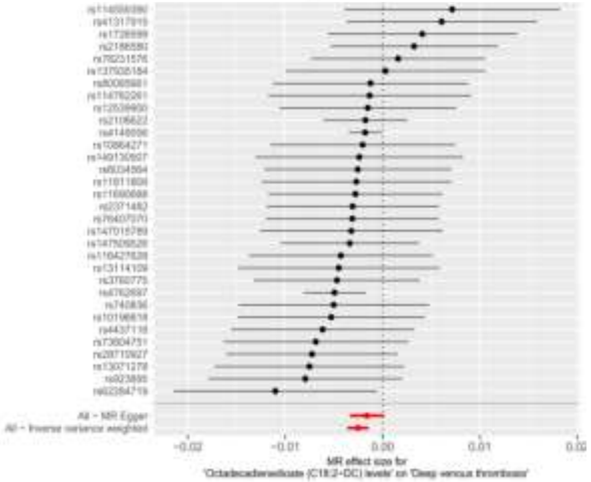

Supplementary file 11:

Visualization of MR result for hosphatidylcholine (18:0_18:2) levels on Deep venous thrombosis (DVT).

Visualization of the MR Results (*Phosphatidylcholine (18:0_18:2) levels on DVT*)

Scatter plot

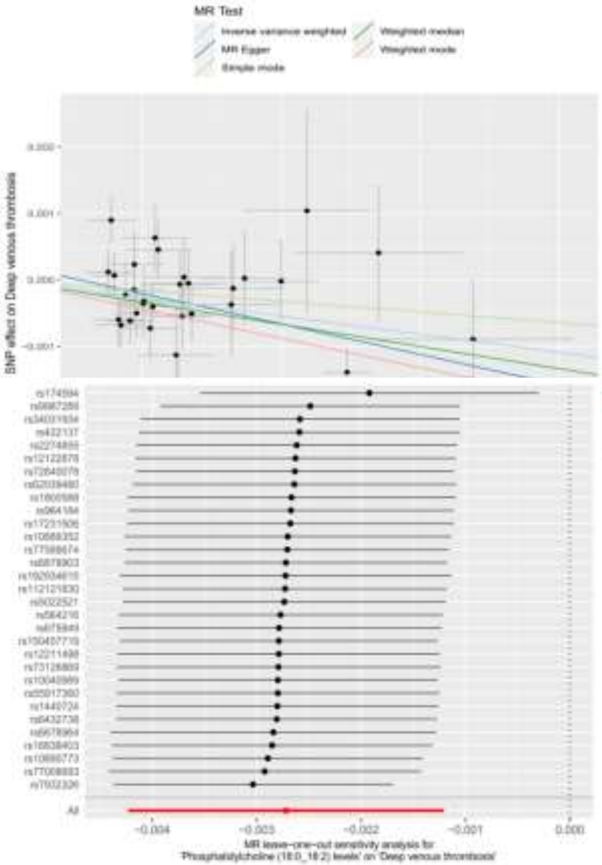

Funnel plot

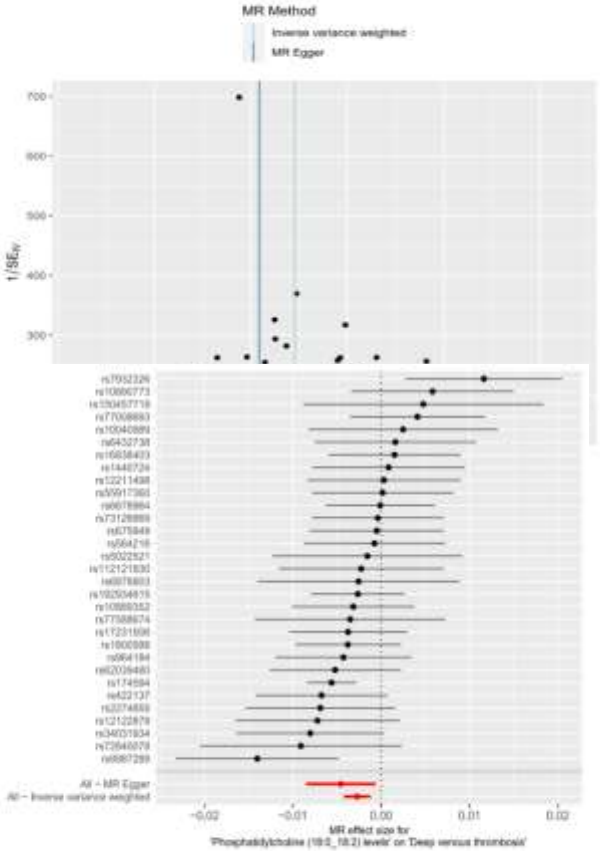

Leave-one-out analysis

Forest plot
